# The effect of the surgical approach and cochlear implant electrode on the structural integrity of the cochlea in human temporal bones

**DOI:** 10.1038/s41598-022-21399-7

**Published:** 2022-10-12

**Authors:** Saad Jwair, Huib Versnel, Robert J. Stokroos, Hans G. X. M. Thomeer

**Affiliations:** 1 Department of Otorhinolaryngology and Head & Neck Surgery, University Medical Center Utrecht, Utrecht University, Heidelberglaan 100, P.O. Box 85500, 3508 Utrecht, GA The Netherlands; 2 UMC Utrecht Brain Center, Utrecht University, Utrecht, The Netherlands

**Keywords:** Neuroscience, Auditory system, Cochlea, Inner ear

## Abstract

Cochlear implants (CI) restore hearing of severely hearing-impaired patients. Although this auditory prosthesis is widely considered to be very successful, structural cochlear trauma during cochlear implantation is an important problem, reductions of which could help to improve hearing outcomes and to broaden selection criteria. The surgical approach in cochlear implantation, i.e. round window (RW) or cochleostomy (CO), and type of electrode-array, perimodiolar (PM) or lateral wall (LW), are variables that might influence the probability of severe trauma. We investigated the effect of these two variables on scalar translocation (STL), a specific type of severe trauma. Thirty-two fresh frozen human cadaveric ears were evenly distributed over four groups receiving either RW or CO approach, and either LW or PM array. Conventional radiological multiplanar reconstruction (MPR) was compared with a reconstruction method that uncoils the spiral shape of the cochlea (UCR). Histological analysis showed that RW with PM array had STL rate of 87% (7/8), CO approach with LW array 75% (6/8), RW approach with LW array 50% (4/8) and CO approach with PM array 29% (2/7). STL assessment using UCR showed a higher inter-observer and histological agreement (91 and 94% respectively), than that using MPR (69 and 74% respectively). In particular, LW array positions were difficult to assess with MPR. In conclusion, the interaction between surgical approach and type of array should be preoperatively considered in cochlear implant surgery. UCR technique is advised for radiological assessment of CI positions, and in general it might be useful for pathologies involving the inner ear or other complex shaped bony tubular structures.

## Introduction

Worldwide, the prevalence of hearing loss is increasing, with currently more than half a billion people with disabling hearing loss^1^. Severe hearing loss is recognized as an important health issue that can lead to depression, insecurity, language development delay and social isolation^2^**.** Severe to profound hearing loss can be treated with a cochlear implant (CI)^2^. A CI converts sound into electrical current pulses that stimulate the auditory nerve. The CI bypasses affected and degenerated sensory receptor cells. Outcomes of CIs have improved tremendously in the past 45 years, drastically changing the perspective for hearing-impaired patients^2^.

However, understanding speech in background noise, and musical melody perception, are challenging or impossible for most CI recipients^3^. In most cases, severely hearing-impaired patients have some residual hearing on the lower frequencies^4^. The preservation of this residual hearing (i.e. hearing preservation) might help CI patients with speech perception^5–8^. In addition, selection criteria for CI are difficult to define, and they differ among countries^9^. Importantly, some hearing-impaired patients fail to achieve satisfactory results with either of the treatment options for a considerable time^9^. On the one hand they achieve unsatisfactory results with hearing aids, but on the other hand fall below threshold for a CI because their hearing is too good. Considering that hearing deteriorates with increasing age, those patients will likely meet the selection criteria for a CI over time^10^. Broadening medical criteria for a CI, however, would permit these patients to receive a CI at an earlier time point. In addition, it would allow for CI treatment of patients with severe tinnitus but relatively good hearing^11^. A major hurdle for broadening these medical criteria can be overcome by preserving the residual hearing through means of limiting structural trauma to the cochlea during cochlear implantation^12^. In recent years, the development of robot-assisted approaches and array insertions are being explored to this end^13,14^. In addition, limiting trauma opens the way for future developments relying on cochlear structure preservation, e.g. use of corticosteroids or neurotrophin eluting CIs, or hair cell regeneration^15,16^. Minimizing cochlear trauma during implantation can also reduce fibrosis and ossification on the long term making potential reimplantations easier to conduct^17^. This latter aspect is especially relevant in pediatric patients, as they have increased risk for reimplantation during their lifetime due to malfunctions or necessary upgrades^18^.

**Figure 1:**
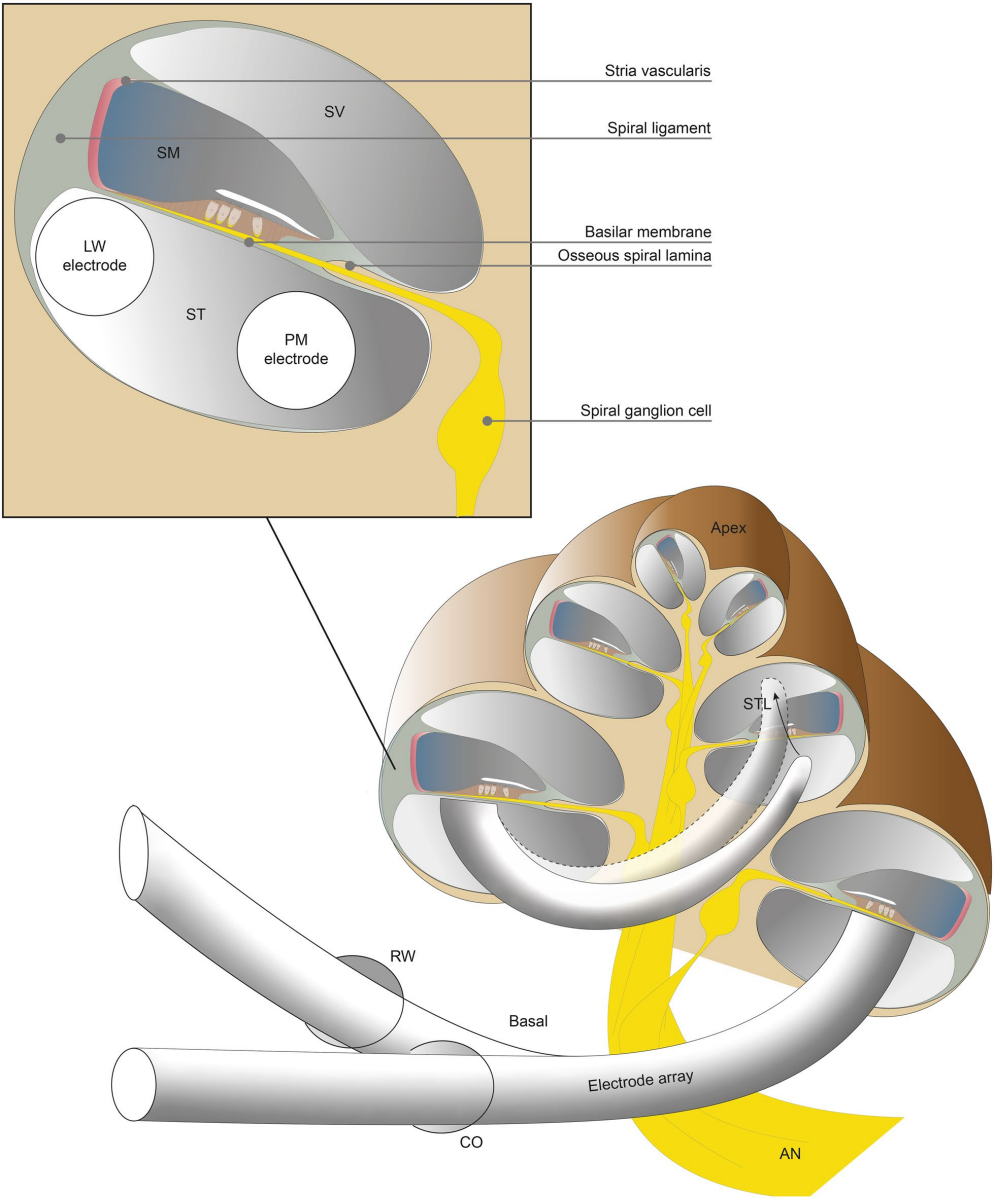
A cross section of the cochlea is depicted with an implanted electrode array. The electrode array is implanted in the scala tympani, using either the round window or a small hole in the cochlea (cochleostomy) for entry. The array follows the spiral curvature of the cochlea from the base of the cochlea towards the apex. Arrays usually reach at least around one turn and half, depending on the exact length of the array. Perimodiolar arrays are positioned more towards the spiral ganglion cells of the auditory nerve and beneath the osseous spiral lamina, and in contrast, lateral wall arrays are positioned laterally towards spiral ligament and beneath basilar membrane. In some cases the array can translocate during insertion (i.e. STL) from ST to SV or SM, which is detrimental for the structures that lie in between. *RW* round window, *CO* cochleostomy, *AN* auditory nerve, *STL* scalar translocation, *ST* scala tympani, *SV* scala vestibule, *SM* scala media, *LW* lateral wall, *PM* perimodiolar.

Recent evidence shows that scalar translocation (STL) of electrode-arrays (in short: arrays), which leads to severe trauma, is frequently occurring in CI surgery^19^. Normally, the array is inserted into the scala tympani (ST), however in some cases the array translocates (i.e. STL) to scala media (SM) or scala vestibuli (SV), as illustrated in Fig. 1.

STL avoidance was on the forefront of development by manufacturers of newer versions of two type of arrays, lateral wall (LW) and perimodiolar (PM)^20^, see Fig. 1. Both array types are commonly used in medical practice. LW arrays, or straight arrays, were used initially and have been continuously developed to be less traumatic. They are smaller in diameter nowadays, more flexible and have more rounded tips compared to previous generations. PM arrays were developed as an alternative to LW arrays to achieve a position closer to the modiolus^21^. They are precurved in order to follow the spiral shape of the cochlea. These arrays need to be straightened before implantation, to which both stylet and sheath based methods exist. The stylet and sheaths are removed after achieving insertion in the basal part of the cochlea during insertion, allowing the array to curl against cochlear modiolus and reducing the electrode-neuron distance for electrical stimulation. This in theory achieves better frequency resolution by lessening the spread of excitation across electrodes. PM arrays, although smaller than previous generations, are in general larger in diameter than the latest LW arrays, probably because of aforementioned methods needed to insert these arrays into the cochlea^20^.

The surgical approach for insertion might also be an important factor in STL. Two approaches are mostly used for array insertion^22^. The round window (RW) approach is conducted, after drilling of the bony overhang to expose the RW membrane, through a slit like opening in the RW membrane for entry in the cochlea. In contrast, a cochleostomy approach (CO) uses a burr-hole opening in the cochlea, anterior and inferior to the RW membrane, for entry (Fig. 1). Another approach, the extended round window, is a combination of RW and CO approach. The RW and CO approaches lead to different insertion angles that likely influence intra-scalar positioning of arrays^23^. While the conventional CO approach is still widely used, CI surgeons currently gravitate towards use of RW approach, as it is perceived to be less invasive to cochlear structures^24–26^.

To this date, no study has addressed the effect of both surgical approaches and the latest types of arrays on STL. Previous studies have investigated both variables separately regarding trauma severity. Studies showed that RW approach leads to less intracochlear trauma than CO approach^27,28^, although a systematic review showed inconclusive results^29^. In addition, another systematic review showed that LW arrays induce less severe cochlear trauma compared to PM arrays^19^. Possible interaction between electrode choice in relation to surgical approach have not been systematically studied thus far. An example of such an effect is the smaller size of entry to the cochlea of a RW approach, compared to CO, possibly leading to more friction and trauma during PM array insertion than insertion with a LW array.

Based on above-mentioned findings in literature, our hypothesis is that the combination of RW approach with LW array leads to the least severe cochlear trauma in the form of STL. To this end, we designed a temporal bone experiment with fresh frozen cadaveric heads investigating the four commonly used combinations of CO or RW with LW or PM. In addition, the diagnostic value of CT imaging using the most common radiological assessment option for STL was compared to a CT scanning protocol with curved multiplanar reconstructions.

## Results

### Histological analysis of scalar translocation

**Figure 2:**
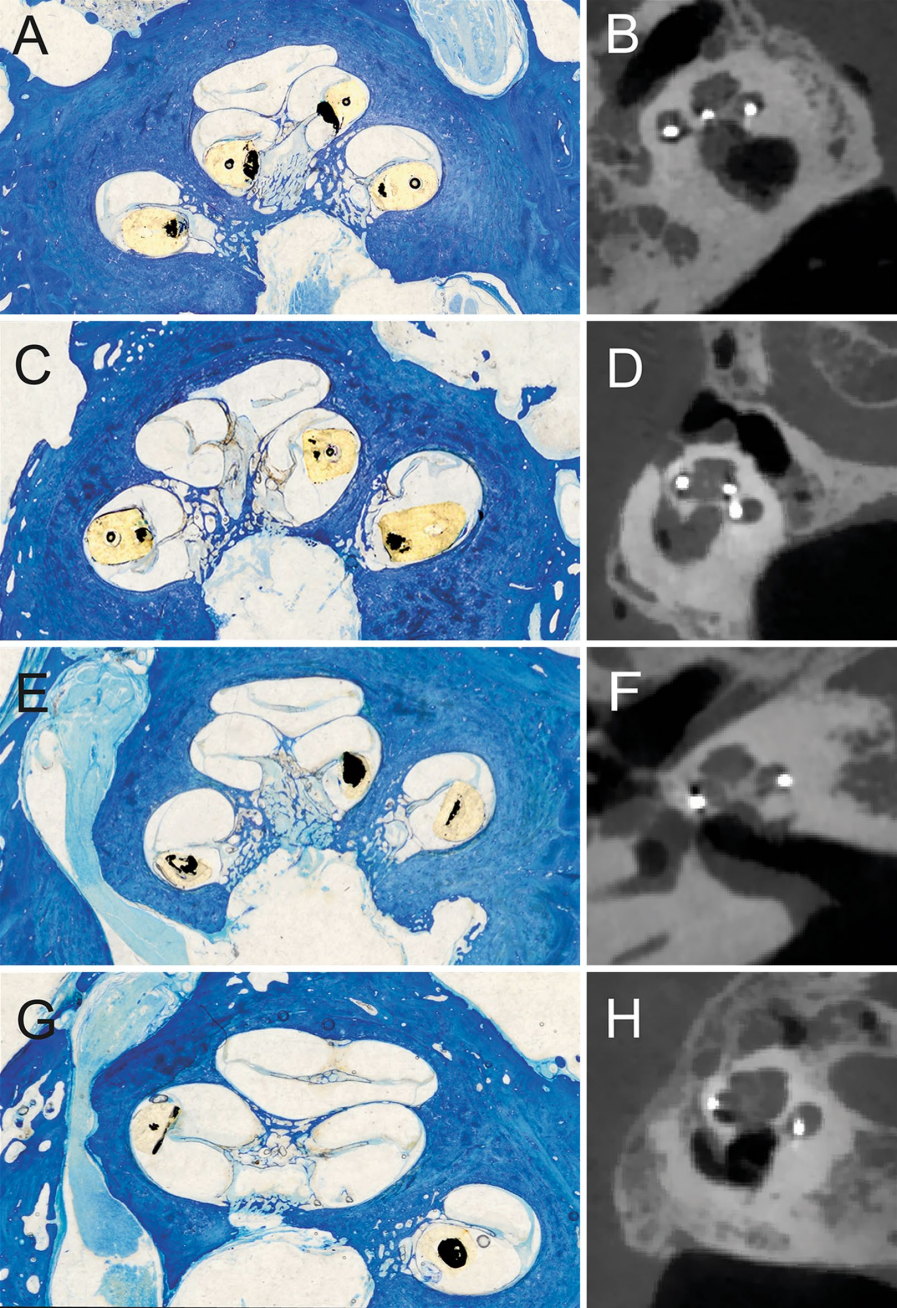
Histological modiolar plane sections and corresponding CT images were compared. Similar array positions were observed between histology and radiology. In (**A,B**) non-STL PM array. In (**C,D**) non-STL LW array. In (**E,F**) STL PM array. In (**G,H)** STL LW array. Note: the diameter of the array was increased 30–40% due to swelling of the silicon layer after processing with butyl methacrylate. Contrast of array was increased for visibility reasons.

Thirty-two samples were sectioned and used for assessment of STL. One sample was excluded, because the sections were not cut in the midmodiolar plane (i.e. no midmodiolar sections were available). Careful analysis comparing histology and radiology showed no signs of swelling influencing STL outcomes. The radiological images were acquired < 1 h after implantation, and before histology. In Fig. 2, the array position of the midmodiolar histological sections and corresponding CT images were compared for a case with non-STL PM array (Fig. 2A,B), STL PM array (Fig. 2C,D), non-STL LW array (Fig. 2E,F) and STL LW array (Fig. 2G,H). The positions in histological and radiological images were similar: array swelling due to histological processing induced no change of array position or severe trauma to cochlear structures, although minor not visible trauma due to swelling cannot be excluded.

In total, 12 out of 31 arrays (39%) were fully located in ST, and 19 out of 31 arrays (61%) had at least one electrode in either SV (n = 12) or SM (n = 7). Very similar outcomes were observed between two surgeons: 7/11 (63%) and 12/20 (60%) had at least one electrode in either SM or SV (i.e. STL) for respectively HT and SJ.

**Figure 3:**
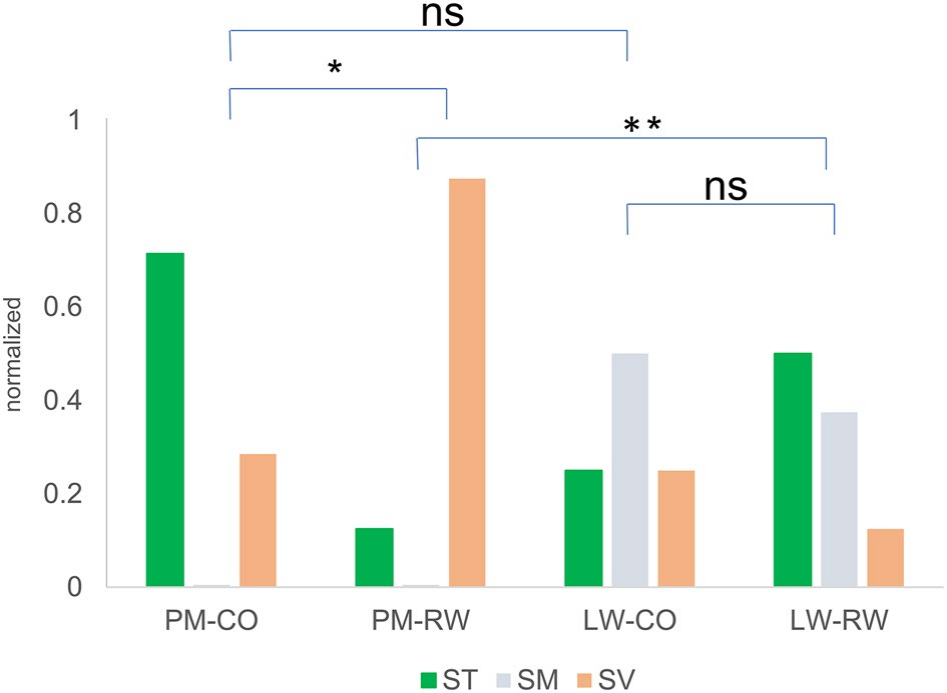
Group comparison of array scalar positioning. Scala media or scala vestibuli position is assigned if at least one electrode-contact was located in the respective compartment. *p < 0.05, **p < 0.01. *ST* scala tympani, *SM* scala media, *SV* scala vestibuli.

Figure 3. shows the scalar position distribution for the four groups. The RW approach with PM array had a STL rate of 87% (7/8), CO approach with LW array 75% (6/8), RW approach with LW array 50% (4/8) and CO approach with PM array 29% (2/7). This is a significant difference (p = 0.016, Fisher’s Exact test). The PM-CO group had the smallest STL rates, while the PM-RW group had the largest STL rates. Comparing these two PM groups shows a significant difference (p = 0.041, Fisher’s Exact test). Comparing the RW groups we also see a significant difference (p = 0.01, Fisher’s Exact test). No statistical differences were observed between array types with CO approach (i.e. LW-CO vs. PM-CO) and between surgical approaches with LW array (i.e. RW-LW vs. CO-LW).

**Figure 4:**
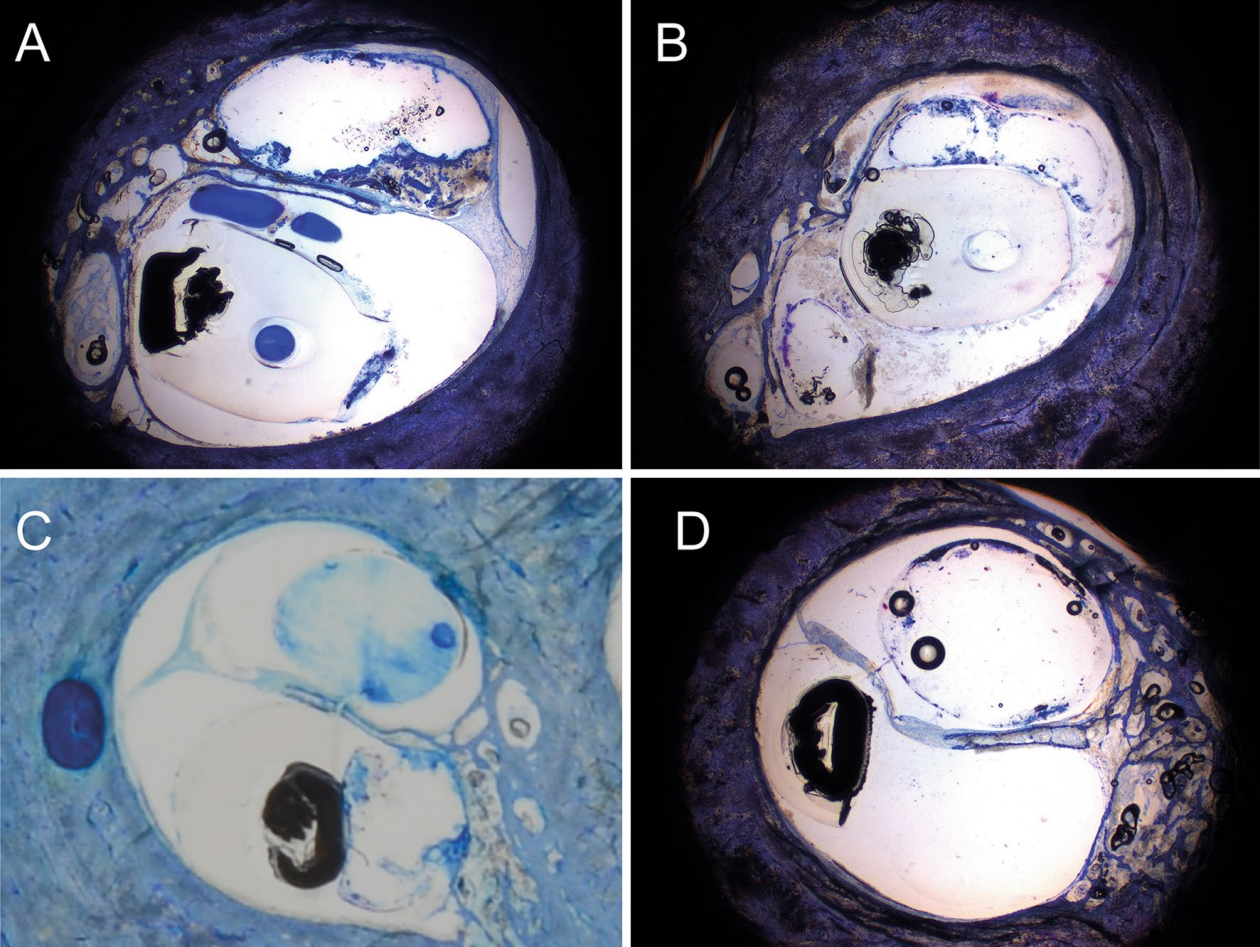
Histological modiolar plane sections. (**A**) non-STL PM array. (**B**) STL array with fracture of osseous spiral lamina. (**C)** non-STL LW array. (**D**) STL LW array to scala media with displacement of stria vascularis, basilar membrane and spiral ligament.

The two types of arrays have different positions in ST (as schematically depicted in Fig. 1). On the one hand, the PM array is located medially towards Rosenthal’s canal and beneath the osseous spiral lamina (Fig. 4A). On the other hand, the LW array is, as intended, located more laterally towards the stria vascularis (Fig. 4C). When STL occurred, the kind of inflicted trauma differed between LW and PM arrays. In PM arrays, if translocated, the array always fractured the osseous spiral lamina (Fig. 4B). In contrast, in LW arrays, SM was in several cases (n = 7/10, 70%) severely crushed and pushed towards SV, including stria vascularis and basilar membrane trauma but without osseous spiral lamina fracture (Fig. 4D).

**Figure 5:**
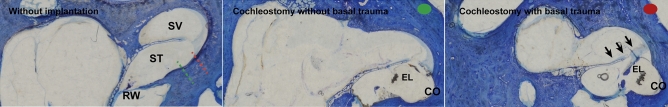
Histological modiolar plane sections. On the left a non-implanted basal turn of the cochlea. The green and red dotted lines depict angles of cochleostomy site of respectively the middle and right image. Green dotted line represents an antero-inferiorly placed cochleostomy, the red dotted line represents slight displacement of cochleostomy to anterior. In middle image, corresponding to the green line, no basal trauma is observed. In right image, corresponding to the red line, basal trauma is observed: displacement of stria vascularis, basilar membrane and osseous spiral lamina resulting in a crushed scala media compartment. *RW* round window, *ST* scala tympani, *SV* scala vestibuli.

Cochleostomy burr hole that is too anteriorly placed can lead to trauma around the site of cochleostomy, thus in the very most basal part of the cochlea, as illustrated in Fig. 5. This trauma affects the osseous spiral lamina, and can result in direct insertion into SV, resulting in an unintended complete insertion of the array into SV. In total, of 16 insertions using the CO approach, four cases had such trauma. In two of these cases, the array was indeed completely located in SV, and in the other two cases the arrays were completely located in ST. In remainder of the CO cases no direct basal trauma around the CO site could be objectified.

### Radiological analysis of scalar translocation

**Figure 6:**
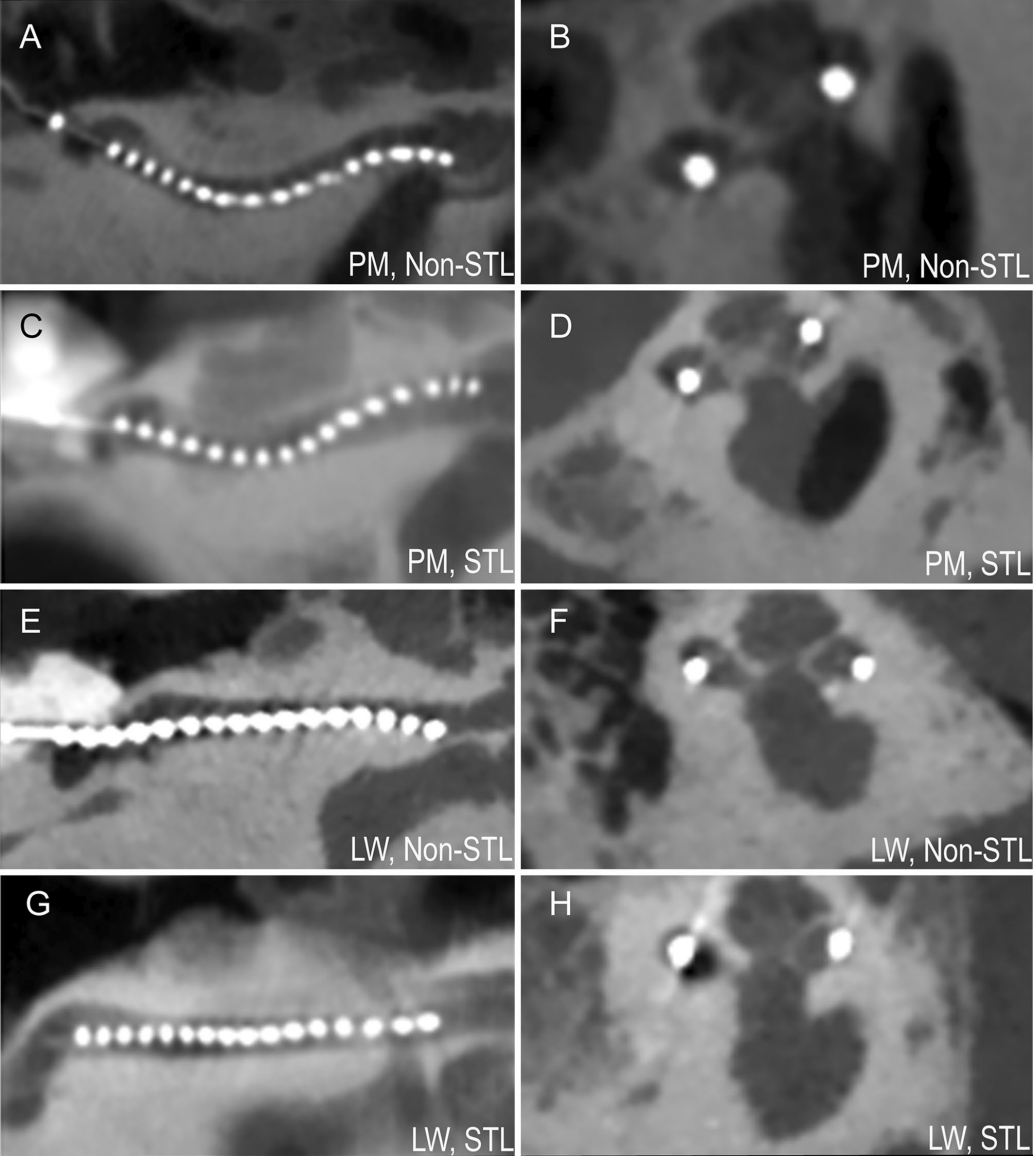
Assessment examples between uncoiled cochlear reconstructions (on the left) and conventional multiplanar reconstructions (on the right). In (**A**) the PM array is neatly following the scala tympani, which is located in lower half of the uncoiled cochlea. In (**C**), however, clear kinking of the PM array results in STL from scala tympani to scala vestibuli. This difference of PM non-STL vs. STL is also seen in conventional reconstructions in (**B,D**), with the array, the white dots, located in STL case of (**D**) more towards the upper half of the cochlea than in (**B**). In (**E**) the LW array follows the scala tympani without interruption, in contrast in (**G**), the LW array shows an subtle, but still clear kink towards scala vestibuli (i.e. STL). The different position of the LW array is difficult to observe in conventional reconstructions, see (**F**) vs. (**H**).

The 32 ears were imaged with CB-CT scanner. All scans were of sufficient quality, and thus included in this study. For STL analysis, two types of reformatted CB-CT scans were used, uncoiled cochlear reconstructions (UCR) and multiplanar reconstructions (MPR), for both of which four example cases are illustrated in Fig. 6, with the UCR on the left and the MPR on the right. In cases with the PM array a STL event could be easily identified as a jump of the array to SV (see Fig. 6C vs. 6A). In contrast, LW arrays can be situated in an intermediate position at SM, and therefore show a more subtle scalar jump (see Fig. 6G vs. 6E).

In the MPRs, the normal non-STL positions of LW vs PM arrays are clearly different (Fig. 6B vs. 6F). Both these positions are in ST, with the PM array being closer to cochlear modiolus, and the LW array lying towards the lateral wall of the cochlea. Figure 6D shows that an electrode of the PM array is located in the upper half of the cochlea, therefore located most likely in SV, clearly different from non-STL (Fig. 6B). In contrast, for LW arrays, it is more challenging to differentiate between STL and non-STL arrays (see Fig. 6H vs. 6F). The array is located in both cases laterally and towards the SM, with a subtle difference showing the non-translocated array located lower than the translocated array. 

**Figure 7:**
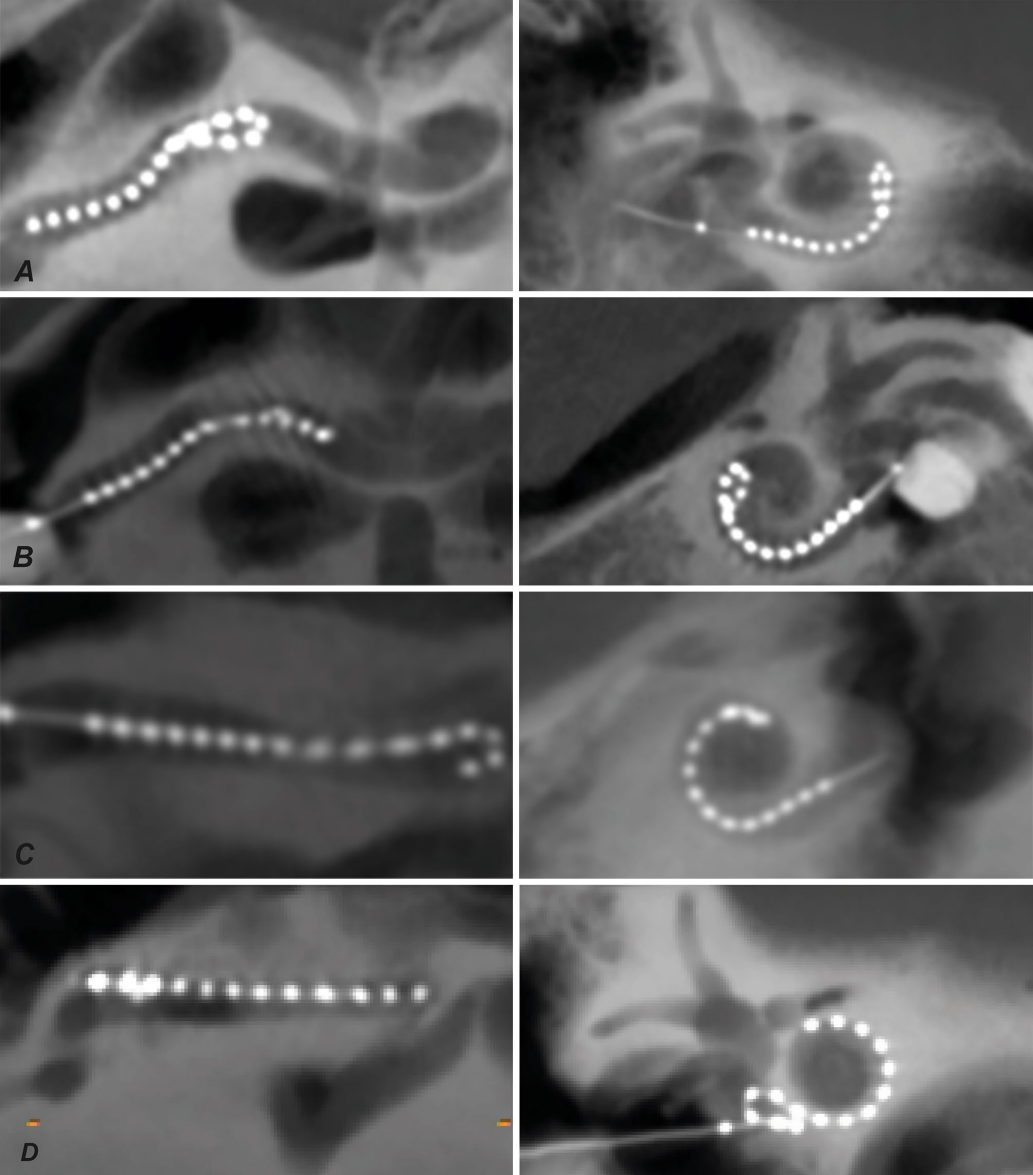
Four fold-overs were observed, depicted on the left with uncoiled cochlear reconstructions and on the right with conventional reconstructions. In (**A–C**) a PM array was used. Two tip fold-overs occurred at similar position (see **A,B**), approximately at insertion depth of 180°, while the other tip fold-over occurred deeper at around 270° (**C**). Finally, in (**D**) a case with a basal fold-over had occurred using the LW array. In both reconstruction techniques the fold-overs were clearly seen. *PM* perimodiolar, *LW* lateral wall.

Array fold-over occurred in four cases (4/32 = 12%), illustrated in Fig. 7 with for every case a UCR (left) and oblique coronal plane image of the cochlea (right). In three cases a tip fold-over had occurred with a PM array (A–C). Two tip fold-overs occurred at similar position (see Fig. 7A,B), approximately at insertion depth of 180°, while the other tip fold-over occurred deeper at around 270° (Fig. 7C). In the first two cases tip fold-over had occurred most likely because of a too shallow insertion of the array with stylet. After removing the stylet, the array bumped against the modiolar wall, rather than following the curvature of the cochlear duct. For the third case, with a deeper insertion, it seems that the electrode contacts were slightly tilted away from the modiolar wall. Finally, one case with a LW array had a fold-over in the basal end of the cochlea (Fig. 7D). In this case, the surgeon continued array insertion to reach full insertion even though resistance occurred early during insertion. This was an exception: normally array insertion is not continued when resistance is encountered, however in this case the resistance occurred at the basal turn (i.e. very shallow insertion).

### Inter-observer and inter-method agreement

Table 1 (left side) shows the inter-observer agreement for both UCRs and MPRs. The agreement between the two assessors for UCR images was very high: 91%, 29 out of 32, had the same score (к = 0.85). In two out of three cases the two assessors disagreed whether it was SV or SM, thus they agreed on STL, resulting in an agreement score of 97% for STL. The assessors were not in agreement regarding occurrence of STL for just one case, which had a tip fold-over according to histology. The assessors were in agreement when using MPRs for 22 out of 32 cases, resulting in an inter-observer agreement score of 69% (к = 0.45). Two of the ten cases of disagreement had tip fold-overs. For the remaining cases, the assessors mostly disagreed regarding LW arrays (6 out of 8, 75%). The two к values differed significantly (z value 2.01; p = 0.04).

**Table 1:** Scalar translocation event evaluation after cochlear implantation (n = 32)^⁑^.

Inter-observer agreement UCR*: reviewer 1 vs. reviewer 2		Inter-observer agreement MPR^#^: reviewer 1 vs. reviewer 2		Inter-method agreement: CB-CT vs. Histology			
				UCR^†^ vs. Histology^§^		MPR^†^ vs. Histology^§^	
Observed agreement	к^¶^	Observed agreement	к	Observed agreement	к	Observed agreement	к
91%	0.85	69%	0.45	94%	0.89	74%	0.57

⁑scalar translocation event: minimal of one electrode-contact in scala vestibuli or scala media, including direct scala vestibuli insertions; *UCR uncoiled cochlear reconstruction CB-CT images; ^#^MPR axial and sagittal multiplanar reconstructions; ^†^if observations by reviewer 1 and 2 were different, consensus was achieved by final decision of reviewer 3; ^§^based on observations of reviewer 3; ^¶^magnitude of kappa coefficient: < 0: poor, 0.00–0.20 = slight, 0.21–0.40 = fair, 0.41–0.60 = substantial, 0.81–1.00 = excellent agreement.

Table 1 (right side) also shows inter-method agreements between radiological assessment options and histology. The inter-method agreement between UCR and histology was very high: 94% (к = 0.89). In two cases the histology did not match the UCR outcome. In one of these cases a tip fold-over had occurred, which was according to histology a non-STL insertion and was rated in UCR by the assessors as STL in SV. The inter-method agreement between conventional MPR assessment and histology was lower: 74% (к = 0.57), almost reaching a statistically significant difference (z value − 1.81; p = 0.06). In half of the wrongly assessed cases (4 out of 8), differentiating between SM and SV position proved to be difficult. In addition, three STs proved to be false positive (i.e. translocated according to histology), and one STL case was false negative (non-translocated).

### Insertion depth

Insertion depth angles were similar for the four groups when assessing all implanted arrays, with group means ranging from 322 to 374 degrees (Table 2). Three more analyses were performed (see Table 2). (1) Excluding the four tip fold-over cases, array type and surgical approach had significant effect on insertion depth (ANOVA, p = 0.01 and p = 0.046 respectively). PM arrays reached higher insertion depths than LW arrays (mean 392° vs. 342°), and CO approach reached higher depths than RW approach (mean 387° vs. 348°). (2) In addition, six arrays had electrodes outside the cochlea, all LW arrays, ranging from 11 to 15 inserted electrode-contacts (from 16 electrode-contacts in total). Excluding these not fully implanted arrays still shows a main effect of array type (ANOVA, p = 0.01), favoring PM arrays with higher insertion depths (mean 402° vs. 370°). (3). Furthermore, according to the manufacturer, not only the functional 16 electrodes-contacts should be inside the cochlea, but also the stop non-functional electrode-contact should be at RW or CO site. For 11 arrays, all electrodes were inside the cochlea, however, the stop electrode was 1–2 mm outside RW or CO, resulting in a total of 11 full insertions according to the manufacturer. No effect of array type or surgical approach on insertion depth was seen in this last analysis.

**Table 2:** Insertion depth angles.

Group	Mean PM-CO (n)	Mean PM-RW (n)	Mean LW-CO (n)	Mean LW-RW (n)	Main effect approach	Main effect array	Interaction
All	374 (8)	357 (8)	354 (8)	322 (8)	p = 0.31	p = 0.25	p = 0.74
(1) Minus TFs	410 (6)	375 (7)	365 (7)	322 (8)	**p = 0.046**	**p = 0.01**	p = 0.83
(2) Minus TFs and < 16E inserted	410 (6)	395 (6)	383 (5)	357 (5)	p = 0.09	**p = 0.01**	p = 0.63
(3) Full insertion (according to manufacturer)	412 (3)	398 (4)	407 (3)	372 (1)	p = 0.29	p = 0.46	p = 0.92

Significant values are in bold.

*TF* tip fold-over, *PM* perimodiolar, *LW* lateral wall, *CO* cochleostomy, *RW* round window, *E* electrode-contacts.

**Figure 8:**
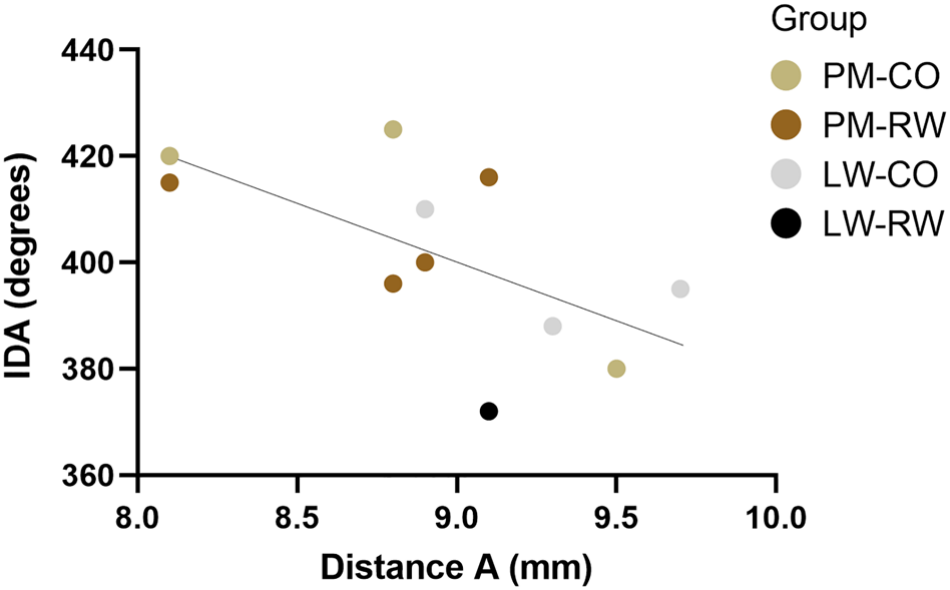
The insertion depth angles and corresponding cochlear distance A are plotted of the 11 full insertions according to the manufactures guidelines. An inverse correlation is observed between insertion depth angle and distance A (R^2^ = 0.39, p < 0.05). Distance A is an indirect measure of cochlear duct length. *IDA* insertion depth angle, *PM* perimodiolar *LW* lateral wall, *CO* cochleostomy, *RW* round window.

**Figure 9:**
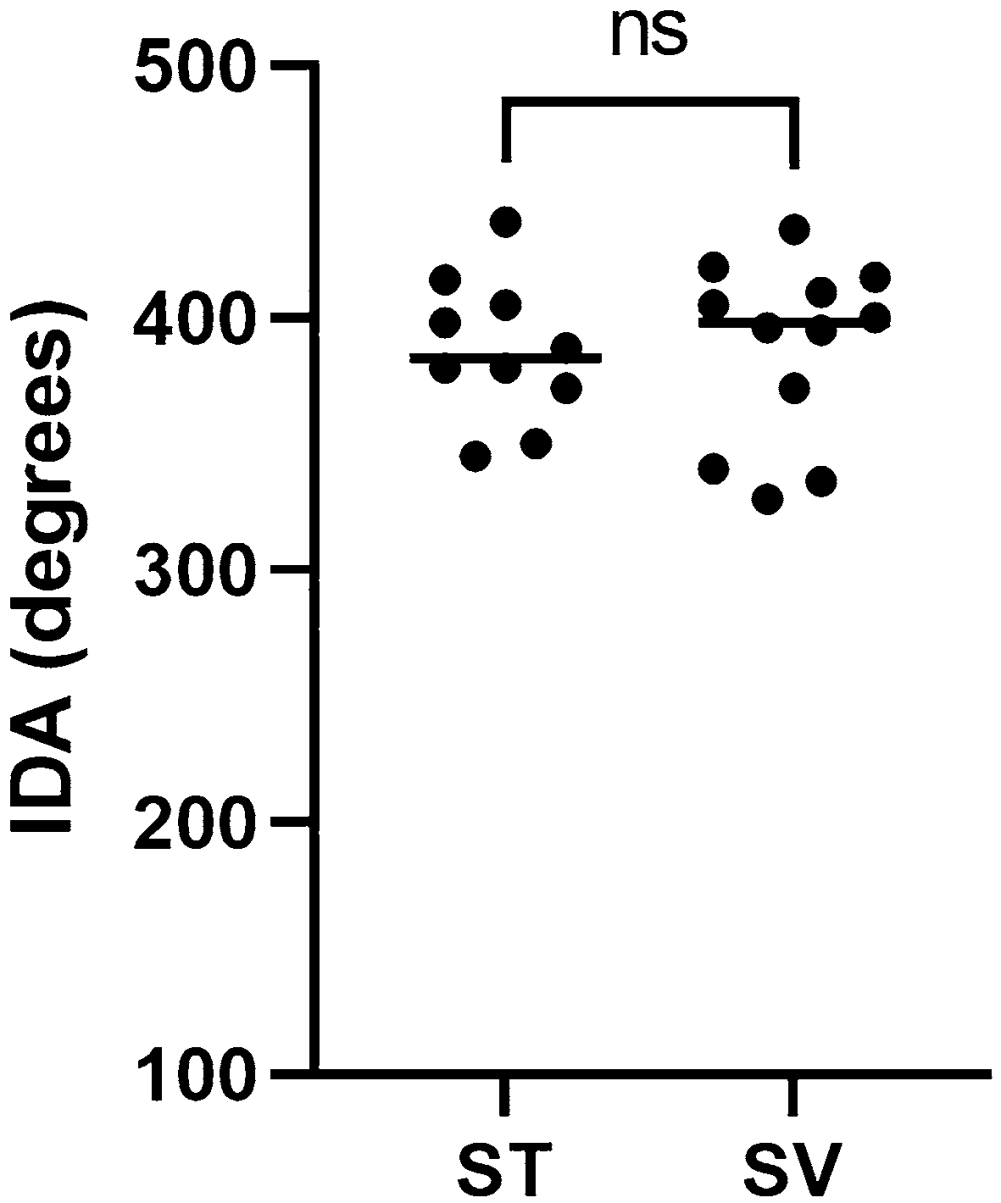
Insertion depth angle is compared between fully scala tympani located arrays and arrays with at least one electrode in scala vestibuli. All insertion depth angles were plotted of arrays with the stop electrode within 2 mm distance from the CO or RW site (n = 22). Median of both groups are shown. *IDA* insertion depth angle, *CO* cochleostomy, *RW* round window.

Regarding these 11 fully implanted arrays we found that insertion depth was inversely correlated with distance A, which reflects the size of the cochlea (R^2^ = 0.39, p = 0.04), i.e. a small cochlea leads to a larger insertion depth angle (Fig. 8). Insertion depth for all 22 arrays that had all 16 electrode-contacts inside the cochlea (see aforementioned analysis 2) does not change when these arrays translocate to SV (unpaired *t* test, p = 0.11; Fig. 9).

## Discussion

We have investigated cochlear implantation in cochleas of fresh frozen cadaveric heads comparing the two types of arrays (LW and PM), and using the two commonly used surgical approaches (CO and RW). We showed that STL, considered as severe cochlear trauma, is frequently occurring and affected by choice of array and surgical approach. In addition, assessment of LW array positioning by conventional CT analysis appeared to be difficult. An adaptation of a reconstruction CT technique using curved reconstructions^30,31^ showed superior results, correlating significantly better with histological outcomes than the conventional technique. This technique, which is readily available in clinical medical care, might be useful for radiological assessment of pathologies concerning the cochlea and vestibular organ, and other pathologies involving complex shaped bony tube-shaped structures (e.g. facial nerve canal integrity assessment in human temporal bone trauma)^32^.

Our study showed a mean STL occurrence rate of 61%, which is higher than shown in a review study with both temporal bones and CI recipients (mean of 18% trauma)^33^. This discrepancy might be due to methodological differences. In this study, implantation was performed solely in human cadaveric heads, not in vivo, which might affect STL outcomes in two ways. Use of human cadaveric heads allows for histological assessment, and therefore more thorough investigation of scalar position^34^. In addition, implantation in human cadaveric heads is more prone to friction and resistance during insertion as dead tissue is less flexible, leading to higher insertion forces^34,35^. Both these factors might explain higher STL rates found in our study than in other studies. Another important factor is difficulty in assessing trauma when LW arrays are used. The LW arrays lie laterally in the scala tympani, and are enveloped by the basilar membrane and spiral ligament. In our study displacement of these structures, albeit partially to scala media, was seen as STL. However, in literature these cases are not always seen as STL, e.g. one study^36^ judges pushing of basilar membrane as minimal insertion trauma (see also^37–39^). The complex anatomy at LW array site might also have led to underestimation of LW array STLs in previous studies, which in most cases relied solely on conventional MPRs for STL diagnosis. The STL rate of our study is more in line with previous studies when accounting for these differences, i.e. without SM cases: our study had STL rate of 39% vs. 42% in another similar histological temporal bone study^34^, and 24% STL rate in review study with live CI recipients^19^.

We showed than if opting for RW approach more STLs were observed with PM arrays than LW arrays. A recent systematic review showed similar results with PM arrays in general (i.e. including other brands) translocating more often than LW arrays when using a RW approach (41% vs. 7%)^19^. Considering also that PM-RW combination leads to more STLs than PM-CO, these findings point to an interaction effect between RW and PM arrays. It is likely that RW and PM array combination leads to more insertion forces, resulting in severe trauma^34,40^. Another study also showed more STLs with PM-RW combination in temporal bones using an older generation PM array^41^. Several factors might be responsible. Firstly, the cochlear hook region may lead to more resistance during insertion^42,43^. The cochlear hook region is directly adjacent to RW, and has a complex anatomical shape with varying width and height along its course^42,43^. The cochlear hook region can be an issue with RW entry, while a CO approach uses a different insertion angle that bypasses largely the cochlear hook region with a more straight insertion approach^27^. Another factor is the size and shape of the RW membrane, which can vary greatly in roundness with sizes ranging from 0.9 to 2.1 mm diameter for the shortest diagonal^43,44^. The cross section of the largest basal part of the PM and LW arrays of Advanced Bionics, used in this study, is approximately the same, around 0.49 mm^2^, with PM arrays having square cross sections and LW arrays having larger flat side and smaller rounded side. The largest part of these cross sections is smaller than the smallest dimensions of the RW membrane (~ 0.7 mm vs. 0.9 mm), and therefore these arrays should fit through the RW membrane. In addition, often the crista fenestra, a bony crest structure within the RW niche, can form an obstacle that further decreases the surface area of the RW membrane^45^. The different shape and varying size of the RW membrane in conjunction with crista fenestrae can be more an issue with the rigid more square cross-sectional shaped PM array that requires a stylet for insertion. Therefore, PM arrays should be used in conjunction with a CO approach. In our study indeed less STLs were observed with PM-CO approach. However, this is contradicted by a study with CI recipients that showed RW approach leading to less STLs than CO approach when opting for PM arrays^28^. The discrepancy with our study could lie in that they investigated different type of PM arrays within their study. In addition, their results were based solely on imaging, making it harder to correctly assess array position. It is also worth considering that studies have shown surgeons often preferring different CO sites^26^. In our study the CO was antero-inferiorly placed relative to RW membrane, in order to avoid the osseous spiral lamina during array insertion and to achieve ST placement. However, if the CO is placed entirely anteriorly, the osseous spiral lamina can form an obstacle for electrode insertion, resulting in an SV translocation. In our study, we have shown that even a small displacement of the CO site can result in trauma to the osseous spiral lamina.

Previous studies showed that shorter arrays lead to better hearing preservation, at least on the short term, and argued that less mechanical trauma occurred with these arrays^46,47^. However, some studies showed that deeper insertion depth is correlated with better speech perception outcomes^48–50^. In contrast, other studies, showed no clear effect of insertion depth on speech perception^51,52^. Both insertion depth and speech perception are influenced by a myriad of factors, making it difficult to investigate this topic accurately, as shown by a relatively recent review with inconclusive results on this subject^53^. In this study, comparable insertion depths for CO and RW approach were found. This is expected as the CO site is very close (< 1 mm) to the RW membrane. Regarding array type we also found comparable insertion depths for the fully implanted arrays, and in line with another study using the same arrays^54^. In addition, we observed that in general, LW arrays were more often not fully inserted, even though full insertion was intended for all implantations. This might indicate that more friction occurred with LW arrays, leading to more detrimental insertion forces. The reason for more friction with LW arrays is unclear, as it might be inherent to the design, or to differences of resistances between modiolar and lateral wall regions. Insertion depth, however, had in general no effect on STL events in our study. A large study of 220 implants in patients showed similar results with no effect of insertion depth on STLs^55^. However, an older study^56^, and a more recent study^57^ showed that deeper insertions are associated with insertion trauma in temporal bones and patients. Although that latter study^57^ is relatively recent, an older generation PM model (Helix of Advanced Bionics) was investigated. Comparing those studies with our study, which uses the latest PM array (Midscala), is therefore somewhat limited. Importantly, in our study, tip fold-overs were only observed for PM arrays, which were always accompanied with STL. This agrees with the general observation that tip fold-overs mainly occur with PM arrays^19^, although, the fold-over rate of CI recipients is reported lower than what we found (i.e. ~ 2% vs. ~ 18%). The difference in rate might be due to several reasons. We used the advance-off-stylet method instead of the insertion tool for inserting PM arrays, however currently no difference between these techniques regarding tip fold-over have been reported. Another factor, related also to the insertion technique, is cochlear implantation experience. Indeed, a previous study has shown that increased experience can lead to less insertion trauma^58^, although that is not always the case^59^. We consider a more likely reason that in prior clinical studies possible fold-overs might have been overlooked. In our study, in one case, as described, the array tip fold-over can be easily missed if not adequately assessed with both axial and coronal views. So even with adequate type of CT, with high resolution and less metal scattering artefacts, a tip fold-over can be overlooked.

Studying intra-cochlear structures in CI patients remains very difficult due to technical limitations of CT-scanners. Metallic ‘bloom’ artifacts can obscure intra-cochlear structures, and CT resolution is still too low to adequately visualize intra-cochlear structures^60^. In the current study whole cadaveric heads were used for scanning, which limits these artefacts, which are more present in isolated temporal bones^61^. Another advantage of scanning the whole cadaveric head is that our images are more similar to images of live patients, and therefore our results are translational to the clinical care. Still, because of the technical limitations, in vivo assessment of array position in the cochlea can only be based on approximate estimates of cochlear structure sites. A study using curved multiplanar reconstructions found similar to our study high interobserver agreement score (93%) for electrode position at 180°^37^. However, a 72% agreement score was found between radiology and histological outcomes. The images in that study had considerable metallic artefacts probably due to scanning isolated temporal bones. This is possibly the reason for the discrepancy with our study that has a higher histological agreement score (94%).

Some studies^62,63^ have focused on other methods to estimate the location of intra-cochlear structures, such as basilar membrane, using both pre- and postoperative images. Computer modeling has been used to estimate basilar membrane position^63^. The model was created using high resolution micro CT images that can depict intra-cochlear structures in cadaveric temporal bones. No data on observer agreement was reported. Another research group used different preoperative micro-CT atlases to find the most fitting atlas for the patient’s cochlea^62^. These atlases are then used as a template for the postoperative CT scans to determine if a translocation had occurred. They found 97% agreement between assessors, and 95% agreement with histology, however, this was based on a small sample size of nine cadaveric temporal bones. These methods are, in contrast to our methods, not readily applicable in every medical center and might be difficult to implement in a large population with great temporal bone anatomy variability. The CB-CT images used in the present study were relative fast and straightforwardly reconstructed, without needing predetermined atlases, using only the postoperative scans. Although the osseous spiral lamina and basilar membrane are not visible on CB-CT scans (nor on conventional CT scanners), highly accurate assessment of scalar position is possible for both type of arrays. 

The benefits of using methyl acrylates are short processing time, high resolution and clear histological sections and low costs, especially when compared to more laborious methods using decalcifying techniques^64^. Although histological processing with methacrylates has been used for many similar studies investigating histological trauma^34,37,62,64–67^, it has its downsides. Methacrylates lead to swelling of the silicone layer of the array, possibly causing (micro) trauma unrelated to cochlear implantation. In our study it was therefore not possible to use grading trauma scale such as the Eshraghi scale^68^. Macroscopic severe trauma, such as STL, is very unlikely to be related to array swelling. The tissue was fixated with formaldehyde before histological processing, and larger structures such as osseous spiral lamina, which is often fractured in cases with STL, are unlikely to be affected by silicone swelling. A previous study, reviewing 21 papers, showed that STL from ST to SV is observed in 85% of the cases with trauma present^33^. In other words, isolated trauma that is less severe than STL is in the minority of CI cases present. Of course, there might be a bias: severe trauma is easier to detect than minor trauma. Still, it is questionable whether a more in depth trauma grading scale is necessary to judge trauma severity of individual CI cases.

## Methods

### Specimen

Fresh frozen human cadaveric heads were obtained from the department of Anatomy in the UMC Utrecht. The specimens were derived from bodies that entered the department of anatomy through a donation program. From these persons written informed consent was obtained during life that allowed the use of their entire bodies for educational and research purposes. These methods are in accordance with UMC Utrecht guidelines, and in accordance to the Dutch law. According to local medical ethical board of UMC Utrecht no additional approvements were required, and thus additional ethical approval was waivered. Ages at death ranged from 59 to 93 years; cause of death was unknown. The specimens were frozen within 48 h postmortem at − 20 °C. The specimen were supplied at random by the prosector for this study. The prosector was not aware of the study purpose. The specimens were thawed 16–24 h before implantation at room temperature (approximately 20 °C). In total, 16 cadaveric heads were bilaterally implanted with an array. The 32 ears were distributed equally over four groups: PM-CO, PM-RW, LW-CO, LW-RW.

### Cochlear implantation surgery

Array insertion was performed according to standard cochlear implantation procedures. After retro auricular incision a mastoidectomy and posterior tympanotomy was performed to reach the middle ear space. Depending on randomization, entry to ST was achieved with either an anteroinferior CO (i.e. relative and < 1 mm to RW membrane) or a pure RW approach. In addition, either a Midscala (PM array; length from electrode-contact at tip to proximal blue marker: 18.5 mm) or SlimJ array (LW array; length from electrode-contact at tip to proximal blue marker: 23 mm) was implanted. These arrays were supplied by the manufacturer (Advanced Bionics^®^). The PM arrays were prior to implantation straightened with a stylet. The arrays have blue markers for gauging the insertion depth of the array. The LW array has one proximal (i.e. basal) blue marker, and it was inserted until this marker reached the CO or RW site. The PM array has in addition to proximal marker also a distal blue marker (i.e. apical): the array with stylet is inserted first until the distal blue marker. Subsequently, the array is pushed over the stylet into the cochlea, while holding the stylet in place (i.e. so called ‘advance off technique’) until reaching the proximal blue marker for full insertion. Duration of array insertion was approximately 20 s. If any resistance was encountered during insertion, the array was carefully and slightly withdrawn, subsequently insertion was continued as normal until full insertion was achieved (if possible). The arrays were fixed with an instant adhesive at posterior tympanotomy site after reaching full insertion. The majority of implantations was performed by the first author (SJ), and the remainder were done by the senior author (HT) who is an experienced otologist. The first author had half year of extensive training in cochlear implantation surgery with fresh frozen cadaveric heads under supervision of senior otologist before commencing these experiments.

### Cone beam CT protocol

All cadaveric heads were scanned within 1 h after implantation. Cone beam CT scanner (3D Newtom, NNT, Italy, 2018) was used for all scans. The tube voltage was 110 kV, with tube charge 30 mC with total scan time of 20 s. The field of view was 8 × 8 cm. Left and right temporal bones were scanned separately. The 3-D volumetric data was reconstructed with isometric 150 µm voxels.

The images were analyzed with software that is supplied by the same CB-CT manufacturer (3D Newtom, NNT, Italy, 2018). Multiplanar reconstructions were made using this software.

### Radiological analysis

**Figure 10:**
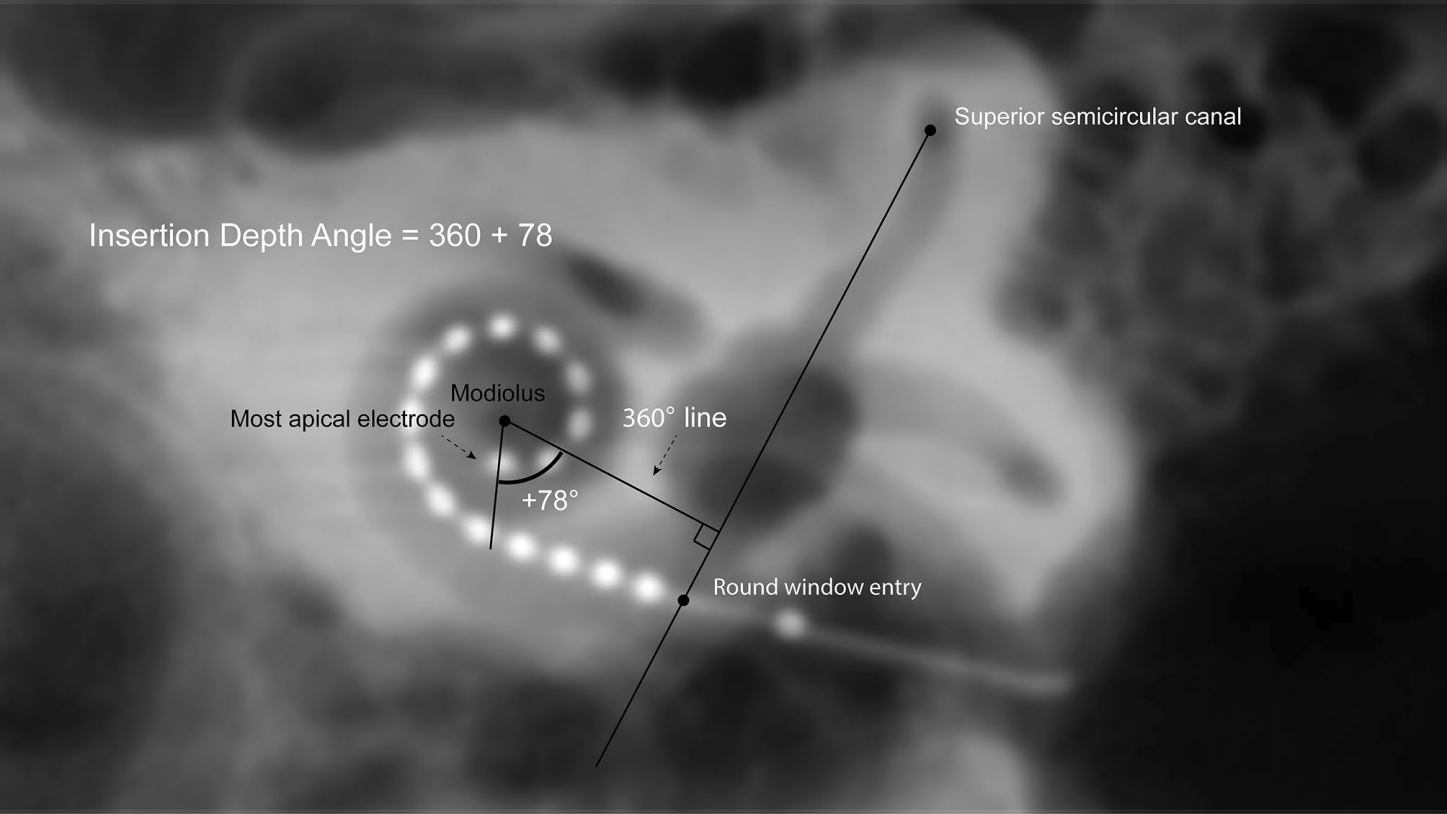
In the cochlear view reconstruction of CB-CT scan both insertion depth angle and distance A can be measured. The 360° line is drawn perpendicular to a line between round window entry and middle of upper part of the posterior semicircular canal. The insertion depth angle is measured by adding 360° to the angle between the apical electrode and the 360° line. Distance A (dashed line), an indirect measure proportional to cochlear duct length, is measured as the length of the line from the point of the array entering the RW or CO, through the modiolus to the contralateral cochlear wall.

Figure 10 illustrates the cochlear view, defined as the plane perpendicular to the basal turn of the cochlea and parallel to the modiolar axis, that was acquired to assess distance A^69^. Distance A is defined as the length of the line between site of entry (CO or RW) through the modiolus to the contralateral wall. This is an indirect measure of cochlear size, proportional to the cochlear duct length. In addition, the Verbist et al. 2010 method was used for determining insertion depth, which was advised in a consensus meeting^70^. To compute the insertion angle and distance A, the images were analyzed with ImageJ software (U. S. National Institutes of Health, USA).

**Figure 11:**
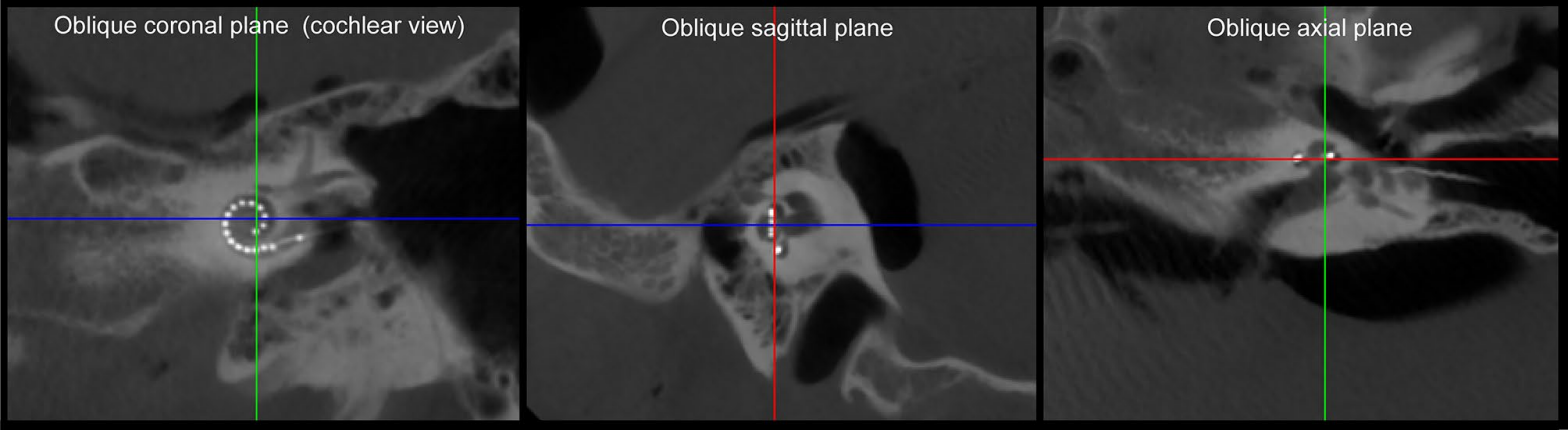
Method for conventional multiplanar reconstructions. The lines in the images represent the position of the shown images relative to the planes. Oblique planes were reconstructed by aligning the red line in sagittal and axial plane to the basal turn of the cochlea, and the blue and green line were aligned to the course of the cochlear modiolus. This resulted in multi reconstructions of three oblique planes: oblique coronal plane, oblique sagittal plane or ‘cochlear view’, and oblique axial plane.

**Figure 12:**
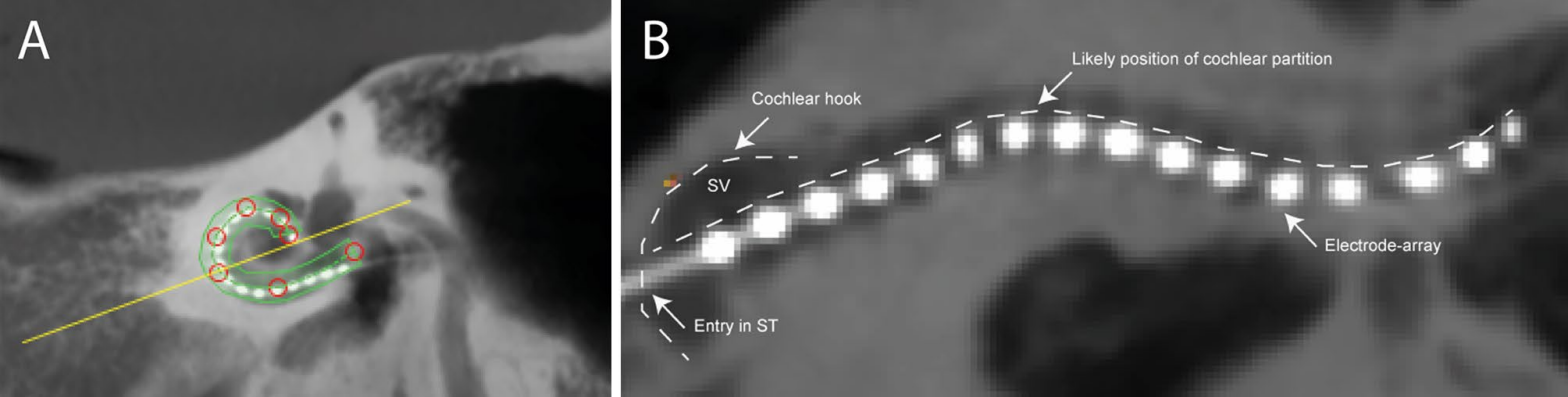
Method for uncoiled cochlear reconstructions (UCR). (**A)** line is drawn along the implanted array in the oblique ‘cochlear view’ reconstruction with 2 mm thickness. The red circles depict the manually selected points that were used to trace the array. The yellow line shows the cross section perpendicular to this tracing line. (**B**) Subsequently, these reconstructions generated a planar two-dimensional image with implanted array, UCR. The cochlear structure is therefore viewed from the side, with upper half being the scala vestibuli (SV) and lower part scala tympani (ST). The first basal segment is characterized by the cochlear hook.

STL was assessed using two types of multiplanar reconstructions. First, after tilting the coronal plane to an oblique plane, the cochlear view image was acquired. Subsequently, conventional multiplanar axial and sagittal reconstructions (MPRs) were created and used for assessment of STL (Fig. 11). Secondly, the cochlea with implanted electrode array was uncoiled using curved multiplanar reconstructions, as introduced by de Seta et al. (2016)^37^ The curved cochlear structure was traced using the trajectory of the electrode array in the cochlear view plane, with a thickness of 2 mm to include also the width of the cochlea (Fig. 12A). Subsequently, these reconstructions generated a planar two-dimensional image, the uncoiled cochlear reconstruction (UCR; see Fig. 12B), which cross-cuts the uncoiled tubular cochlear structure perpendicular along its long axis^37^.

### Histology

The temporal bones with implanted arrays were extracted from cadaveric heads using a large diamond band saw (Exakt-Apparatebau, Norderstedt, Germany), and fixated with formaldehyde (2%). Subsequently, the temporal bones were carefully reduced to small cubes of approximately 1 × 1 × 1 cm^3^ with a smaller diamond band saw (Exakt-Apparatebau, Norderstedt, Germany). We used the posterior tympanotomy site and the internal auditory canal as anatomical boundaries for the region of interest (i.e. cochlea with inserted array). The tissue blocks were dehydrated over two weeks in increasingly higher concentrations of ethanol, starting with 70% ethanol, and finishing with 99%. After dehydration the blocks were embedded for 24 h in butyl methacrylate. Within this period, the blocks were put for 1 h in a vacuum desiccator. After this 24 h period, the blocks were put in an oven at 35 °C for 2 days for polymerization. Modiolar sections of 400 μm thickness were acquired from polymerized blocks using a saw microtome (RMS-16G3; REHA-tech engineering; The Netherlands). The sections were stained with methylene blue and glued with ultraviolet adhesive (Ber-Fix Klebstoffprodukte, Berlin, Germany) on microscope slides. Several non-implanted temporal bones (n = 6) underwent the same procedures. This was done to rule out any structural trauma to cochlear structures arising from the histological procedures (i.e. histological artifacts).

Arrays embedded in butyl methacrylate can increase in size due to swelling of the silicone of the electrode array. Non-implanted arrays were cut in small pieces, and were embedded in butyl methacrylate for 24 h, showing under the microscope (magnification: 2.5 × ) a maximum increase 30–40% in size. This increase was directly visible after embedding. The cochlear tissue was fixated with formaldehyde (first step in tissue processing) before embedding in butyl methacrylate, to make it unlikely that swelling induced secondary damage to cochlear structures such as osseous spiral lamina, stria vascularis and basilar membrane.

### Assessment of scalar translocation

The scalar position of each electrode of the array was assessed both using histology and radiology. STL of an array was noted if at least one electrode was in either SV or in SM. Direct SV insertions were also rated as STL. Histology was used to validate the radiological STL scores.

To assess inter-observer reliability regarding both UCRs and MPRs, two assessors independently assessed occurrence of STL. Assessors were blinded for case number and treatment in order to allow for independent assessment. In addition, the case order was shuffled between the two types of images, to avoid further linkage between UCR and MPR images. A third assessor (SJ) decided the final outcome if the first two assessors disagreed. SJ assessed the histological sections for STL. To assess the inter-method agreement, the final outcomes of the third assessor were compared with histological outcomes. The histological sections were assessed without knowledge of the radiological outcomes of scalar array position.

### Statistical analysis

STL scores based on histology were compared between the four groups using Fisher’s exact test. Insertion depth differences were assessed with ANOVA test. Pearson’s correlations were used to assess relationship between cochlear size and insertion depth angle. Inter-observer agreement was measured as percent agreement between the two assessors (agreement score divided by total number of observation). Similarly, the inter-method agreement between radiological and histological assessments was measured as percent agreement. The 32 observations for this study are sufficient to assess reliability of these agreements with kappa coefficient. This is according to y = 2a^2^ with *a* being 3 (3 outcomes possible: SV, ST or SM), resulting in need for at least 18 observations^71^.

To determine whether the kappa coefficient values significantly differed we used the following formula^72^:

$$z\; = \frac{{\kappa_{1} - \kappa_{2} }}{{\sqrt {\left( {\sigma_{{\kappa_{1} }}^{2} - \sigma_{{\kappa_{2} }}^{2} } \right)} }}$$

κ_1_ and κ_2_ denote the kappa values and σ_1_ and σ_2_ denote the corresponding standard deviations. The p value was calculated two-sided on the assumption of z being a standardized normal distribution.

## Conclusion

We show that the choice for surgical approach to the cochlea should be based on the planned use of type of array, and the other way round: the choice of array type should be based on the surgical approach. Lateral wall arrays were preferred when a round window approach was used, and cochleostomy approach was preferred when a perimodiolar array was used. In addition, we found that conventional CT reconstruction technique can lead to misinterpretation of lateral wall array position. This has probably led to underestimation of lateral wall array translocations in literature. We show for the first time that a relative easy to implement CT reconstruction method can be used in the clinic to accurately assess translocations for both type of arrays, and is herein superior to conventional CT reconstruction techniques. Radiological assessment of pathologies involving the inner ear, and pathologies involving complex shaped bony tubular structures, might also benefit from this technique.

## Data Availability

Data sharing, including full protocol, datasets and statistical codes will be considered upon reasonable request. The corresponding author (SJ) can be contacted to request the data from this study.

## References

[1] Wilson BS, Tucci DL, Merson MH, O'Donoghue GM (2017). Global hearing health care: New findings and perspectives. Lancet.

[2] Carlson ML (2020). Cochlear implantation in adults. N. Engl. J. Med..

[3] Gifford RH, Revit LJ (2010). Speech perception for adult cochlear implant recipients in a realistic background noise: Effectiveness of preprocessing strategies and external options for improving speech recognition in noise. J. Am. Acad. Audiol..

[4] Snels C, IntHout J, Mylanus E, Huinck W, Dhooge I (2019). Hearing preservation in cochlear implant surgery: A meta-analysis. Otol. Neurotol..

[5] Gifford RH (2013). Cochlear implantation with hearing preservation yields significant benefit for speech recognition in complex listening environments. Ear Hear..

[6] Brockmeier SJ (2010). Music perception in electric acoustic stimulation users as assessed by the Mu.S.I.C. test. Adv. Otorhinolaryngol..

[7] Yuksel M, Meredith MA, Rubinstein JT (2019). Effects of low frequency residual hearing on music perception and psychoacoustic abilities in pediatric cochlear implant recipients. Front. Neurosci..

[8] Turner CW, Gantz BJ, Vidal C, Behrens A, Henry BA (2004). Speech recognition in noise for cochlear implant listeners: Benefits of residual acoustic hearing. J. Acoust. Soc. Am..

[9] van der Straaten TFK, Briaire JJ, Vickers D, Boermans P, Frijns JHM (2020). Selection criteria for cochlear implantation in the United Kingdom and flanders: Toward a less restrictive standard. Ear Hear..

[10] Roth TN, Hanebuth D, Probst R (2011). Prevalence of age-related hearing loss in Europe: A review. Eur. Arch. Otorhinolaryngol..

[11] Van de Heyning P (2008). Incapacitating unilateral tinnitus in single-sided deafness treated by cochlear implantation. Ann. Otol. Rhinol. Laryngol..

[12] Santa Maria PL, Gluth MB, Yuan Y, Atlas MD, Blevins NH (2014). Hearing preservation surgery for cochlear implantation: A meta-analysis. Otol. Neurotol..

[13] Caversaccio M (2019). Robotic middle ear access for cochlear implantation: First in man. PLoS ONE.

[14] Torres R (2018). An optimized robot-based technique for cochlear implantation to reduce array insertion trauma. Otolaryngol. Head Neck Surg..

[15] Budenz CL, Pfingst BE, Raphael Y (2012). The use of neurotrophin therapy in the inner ear to augment cochlear implantation outcomes. Anat. Rec..

[16] Smith-Cortinez N (2021). LGR5-positive supporting cells survive ototoxic trauma in the adult mouse cochlea. Front. Mol. Neurosci..

[17] Ishiyama A, Ishiyama G, Lopez IA, Linthicum FH (2019). Temporal bone histopathology of first-generation cochlear implant electrode translocation. Otol. Neurotol..

[18] Lin JW (2010). Characteristics of malfunctioning channels in pediatric cochlear implants. Laryngoscope.

[19] Jwair S (2021). Scalar translocation comparison between lateral wall and perimodiolar cochlear implant arrays—A meta-analysis. Laryngoscope.

[20] Dhanasingh A, Jolly C (2017). An overview of cochlear implant electrode array designs. Hear. Res..

[21] Gstoettner WK (2001). Perimodiolar electrodes in cochlear implant surgery. Acta Otolaryngol..

[22] Jiam NT, Jiradejvong P, Pearl MS, Limb CJ (2016). The effect of round window vs cochleostomy surgical approaches on cochlear implant electrode position: A flat-panel computed tomography study. JAMA Otolaryngol. Head Neck Surg..

[23] Breinbauer HA, Praetorius M (2015). Variability of an ideal insertion vector for cochlear implantation. Otol. Neurotol..

[24] Roland PS, Wright CG, Isaacson B (2007). Cochlear implant electrode insertion: The round window revisited. Laryngoscope.

[25] Gudis DA, Montes M, Bigelow DC, Ruckenstein MJ (2012). The round window: Is it the “cochleostomy” of choice? Experience in 130 consecutive cochlear implants. Otol. Neurotol..

[26] Iseli C, Adunka OF, Buchman CA (2014). Scala tympani cochleostomy survey: A follow-up study. Laryngoscope.

[27] Richard C, Fayad JN, Doherty J, Linthicum FH (2012). Round window versus cochleostomy technique in cochlear implantation: Histologic findings. Otol. Neurotol..

[28] Wanna GB (2014). Impact of electrode design and surgical approach on scalar location and cochlear implant outcomes. Laryngoscope.

[29] Havenith S (2013). Hearing preservation surgery: Cochleostomy or round window approach? A systematic review. Otol. Neurotol..

[30] Achenbach S, Moshage W, Ropers D, Bachmann K (1998). Curved multiplanar reconstructions for the evaluation of contrast-enhanced electron beam CT of the coronary arteries. AJR Am. J. Roentgenol..

[31] Stimpel B (2018). Automated curved and multiplanar reformation for screening of the proximal coronary arteries in MR angiography. J. Imaging.

[32] Kurihara YY (2020). Temporal bone trauma: Typical CT and MRI appearances and important points for evaluation. Radiographics.

[33] Hoskison E, Mitchell S, Coulson C (2017). Systematic review: Radiological and histological evidence of cochlear implant insertion trauma in adult patients. Cochlear Implants Int..

[34] De Seta D (2017). Damage to inner ear structure during cochlear implantation: Correlation between insertion force and radio-histological findings in temporal bone specimens. Hear. Res..

[35] Schuster D, Kratchman LB, Labadie RF (2015). Characterization of intracochlear rupture forces in fresh human cadaveric cochleae. Otol. Neurotol..

[36] Rivas A (2019). A new lateral wall electrode: evaluation of surgical handling, radiographic placement and histological appraisal of insertion trauma. Otol. Neurotol..

[37] De Seta D (2016). 3D curved multiplanar cone beam CT reconstruction for intracochlear position assessment of straight electrodes array. A temporal bone and clinical study. Acta Otorhinolaryngol. Ital..

[38] Sipari S (2018). Cochlear implantation with a novel long straight electrode: The insertion results evaluated by imaging and histology in human temporal bones. Otol. Neurotol..

[39] Du Q, Wang C, He G, Sun Z (2021). Insertion trauma of a new cochlear implant electrode: Evaluated by histology in fresh human temporal bone specimens. Acta Otolaryngol..

[40] Kaufmann CR, Henslee AM, Claussen A, Hansen MR (2020). Evaluation of insertion forces and cochlea trauma following robotics-assisted cochlear implant electrode array insertion. Otol. Neurotol..

[41] Jeyakumar A, Pena SF, Brickman TM (2014). Round window insertion of precurved electrodes is traumatic. Otol. Neurotol..

[42] Avci E, Nauwelaers T, Hamacher V, Kral A (2017). Three-dimensional force profile during cochlear implantation depends on individual geometry and insertion trauma. Ear Hear..

[43] Rask-Andersen H (2012). Human cochlea: anatomical characteristics and their relevance for cochlear implantation. Anat. Rec..

[44] Atturo F, Barbara M, Rask-Andersen H (2014). Is the human round window really round? An anatomic study with surgical implications. Otol. Neurotol..

[45] Angeli RD, Lavinsky J, Setogutti ET, Lavinsky L (2017). The crista fenestra and its impact on the surgical approach to the scala tympani during cochlear implantation. Audiol. Neurootol..

[46] Suhling MC (2016). The impact of electrode array length on hearing preservation in cochlear implantation. Otol. Neurotol..

[47] Causon A, Verschuur C, Newman TA (2015). A Retrospective analysis of the contribution of reported factors in cochlear implantation on hearing preservation outcomes. Otol. Neurotol..

[48] Finley CC (2008). Role of electrode placement as a contributor to variability in cochlear implant outcomes. Otol. Neurotol..

[49] O'Connell BP (2017). Insertion depth impacts speech perception and hearing preservation for lateral wall electrodes. Laryngoscope.

[50] Canfarotta MW (2021). Relationship between electrocochleography, angular insertion depth and cochlear implant speech perception outcomes. Ear Hear..

[51] van der Jagt MA, Briaire JJ, Verbist BM, Frijns JH (2016). Comparison of the Hifocus mid-scala and Hifocus 1J electrode array: Angular insertion depths and speech perception outcomes. Audiol. Neurootol..

[52] Wanna GB (2015). Impact of intrascalar electrode location, electrode type and angular insertion depth on residual hearing in cochlear implant patients: Preliminary results. Otol. Neurotol..

[53] Heutink F, de Rijk SR, Verbist BM, Huinck WJ, Mylanus EAM (2019). Angular electrode insertion depth and speech perception in adults with a cochlear implant: A systematic review. Otol. Neurotol..

[54] Lenarz T, Buechner A, Lesinski-Schiedat A, Timm M, Salcher R (2020). Hearing preservation with a new atraumatic lateral wall electrode. Otol. Neurotol..

[55] O'Connell BP (2016). Electrode location and angular insertion depth are predictors of audiologic outcomes in cochlear implantation. Otol. Neurotol..

[56] Adunka O, Kiefer J (2006). Impact of electrode insertion depth on intracochlear trauma. Otolaryngol. Head Neck Surg..

[57] Zelener F (2020). Relations between scalar shift and insertion depth in human cochlear implantation. Otol. Neurotol..

[58] Aschendorff A (2011). Insertion results for contour and contour advance electrodes: Are there individual learning curves?. HNO.

[59] Kant E, Jwair S, Thomeer H (2022). Hearing preservation in cochlear implant recipients: A cross-sectional cohort study. Clin. Otolaryngol..

[60] Barrett JF, Keat N (2004). Artifacts in CT: Recognition and avoidance. Radiographics.

[61] Guldner C (2012). Artifacts of the electrode in cochlea implantation and limits in analysis of deep insertion in cone beam tomography (CBT). Eur. Arch. Otorhinolaryngol..

[62] Teymouri J, Hullar TE, Holden TA, Chole RA (2011). Verification of computed tomographic estimates of cochlear implant array position: A micro-CT and histologic analysis. Otol. Neurotol..

[63] Wanna GB (2011). Assessment of electrode placement and audiological outcomes in bilateral cochlear implantation. Otol. Neurotol..

[64] Nageris B, Gazit D (1995). Method for embedding temporal bones of rats in methyl-methacrylate. Ann. Otol. Rhinol. Laryngol..

[65] Gstoettner W (1999). Intracochlear position of cochlear implant electrodes. Acta Otolaryngol..

[66] Adunka O (2004). Cochlear implantation via the round window membrane minimizes trauma to cochlear structures: A histologically controlled insertion study. Acta Otolaryngol..

[67] Adunka O, Kiefer J, Unkelbach MH, Radeloff A, Gstoettner W (2005). Evaluating cochlear implant trauma to the scala vestibuli. Clin. Otolaryngol..

[68] Eshraghi AA, Yang NW, Balkany TJ (2003). Comparative study of cochlear damage with three perimodiolar electrode designs. Laryngoscope.

[69] Koch RW, Ladak HM, Elfarnawany M, Agrawal SK (2017). Measuring cochlear duct length—A historical analysis of methods and results. J. Otolaryngol. Head Neck Surg..

[70] Verbist BM (2010). Consensus panel on a cochlear coordinate system applicable in histologic, physiologic and radiologic studies of the human cochlea. Otol. Neurotol..

[71] Landis JR, Koch GG (1977). An application of hierarchical kappa-type statistics in the assessment of majority agreement among multiple observers. Biometrics.

[72] Martens JW, Versnel H, Dejonckere PH (2007). The effect of visible speech in the perceptual rating of pathological voices. Arch. Otolaryngol. Head Neck Surg..

